# What’s Yours Is Mine: Spontaneous Representation and Memorization of Co-Actor’s Goals

**DOI:** 10.3390/bs16050690

**Published:** 2026-04-30

**Authors:** Zhen Li, Jingyin Zhu, Xutao Zheng, Mengting Xu, Jifan Zhou, Mowei Shen

**Affiliations:** 1 Department of Psychology and Behavioral Sciences, Zhejiang University, Hangzhou 310027, China; 3160101331@zju.edu.cn (Z.L.); zhujingyin@oppo.com (J.Z.); 12239008@zju.edu.cn (X.Z.); 21839049@zju.edu.cn (M.X.); mwshen@zju.edu.cn (M.S.); 2 Guangdong Oppo Mobile Telecommunications Corp., Ltd., Dongguan 523860, China; 3 Zhejiang Key Laboratory of Neurocognitive Development and Mental Health, Zhejiang University, Hangzhou 310027, China

**Keywords:** joint memory effect, cooperative activities, visual search, surprise recognition

## Abstract

Joint action involves more than coordinated activity; it is cooperation grounded in shared intentionality, whereby partners represent an activity as something “we” are doing together. This “we-mode” stance should shape attention and memory, making partner-relevant information psychologically significant because it supports a collective goal. Using a joint-search paradigm, we tested whether people automatically attend to and remember partner goals. Pairs of participants searched for targets from different item categories, and trials were successful only when both responded correctly. A surprise recognition test followed the joint-search task assessing memory for the items. Across Experiments 1 (animate stimuli) and 2 (inanimate stimuli), participants showed better recognition of partner-goal items compared to distractors. Participants also showed enhanced attention to partner-goal items in Experiment 2. In Experiment 3, participants completed the same task, and returned three days later for a recognition test first followed by a second joint-search task with switched targets. Participants continued to show superior recognition for partner-goal items, and search efficiency improved after targets switched, indicating that partner-goal was retained over time and supported later cooperation. Together, these findings demonstrate that human cognition supports joint actions over time by organizing attention and memory around what “we” are doing together.

## 1. Introduction

Joint action is one of the most fundamental—and at the same time, theoretically challenging—phenomena of human social life. Joint action refers to a process in which individuals coordinate their actions and mental states in real time (Sebanz et al., 2006). Although superficially similar forms of “acting together” can be observed in other social species, a growing body of research suggests that human joint action is distinctive in its underlying cognitive architecture and normative foundations (Herrmann et al., 2007; Tomasello et al., 2005).

First, humans exhibit a unique form of cooperation that even young (2–3 years old) children can engage in cooperative behaviors with unfamiliar individuals that they never met before, whereas other species, such as gorillas, typically cooperate only with familiar individuals (Marean, 2015; Tomasello, 2009, 2023). Second, humans excel at understanding and representing collective team goals while simultaneously forming individual sub-goals (Searle, 1995). Third, humans can commit to one joint goal while coordinating their actions with others over extended periods, even in the absence of immediate rewards, allowing large groups to pursue complex, long-term objectives collectively (Cheng et al., 2023; Tomasello & Vaish, 2013). Together, these findings implicate deep cognitive roots underlying the unique joint action of humans. Over the past two decades, the theory of shared intentionality has provided a systematic framework for understanding this species-specific structure (M. Bratman, 2014; Gilbert, 2017; Searle, 1990). According to this account, human joint action is not merely a coordination of individual intentions, but is grounded in a shared psychological state characterized by a we-intention: an understanding that “we are doing something together.” In such states, agents not only represent their own goals, but also understand that their partners are oriented toward the same joint goal, and that this orientation is mutually known (Tomasello et al., 2005). This feature has been argued to mark a crucial divide between human joint action and nonhuman animal coordination, which tends to be opportunistic and to dissolve once immediate individual benefits disappear (Rekers et al., 2011).

### 1.1. Imagined-We Model

The theory of shared intentionality has been formalized in the imagined-we model (Tang et al., 2020, 2025), which highlights that representing co-actors’ goals is fundamental for sustaining joint action. Supporting this idea, empirical studies have shown that humans spontaneously represent their partners’ goals during cooperation, even when such representation is not strictly required by the task (Atmaca et al., 2008; Sebanz et al., 2003, 2005; Vesper et al., 2016).

Importantly, joint action often involves role differentiation, and the computational framework of “we-intention” also suggests that individual contributors are replaceable within a shared goal structure. To enable such flexibility, individuals must be potentially prepared to take over others’ roles when necessary (Slors, 2024; Tomasello, 2014). From this perspective, representing a partner’s goal is not merely beneficial for online coordination but also prepares individuals for future role switching in continued cooperation.

Beyond representing goals, humans also demonstrate an automatic tendency to attend to what others are focused on, with their attention often guided by collaborators’ goals (Mundy & Newell, 2007). This shared focus may enhance memory, particularly through processes of encoding and retrieval (Muzzio et al., 2009). Research has shown that memory performance improves when information is cued by others’ gaze or directed by their tasks. This common focus on a collective goal can improve retention of meaningful information, which benefits both immediate collaboration and future coordination efforts. Studies suggest that individuals involved in collaborative tasks often engage in common coding of shared goals (Elekes et al., 2016; Eskenazi et al., 2013). By memorizing information related to the collective objective, individuals retain valuable knowledge about each other’s roles and tasks (Elekes & Sebanz, 2020). This shared memory further supports seamless role replacement, as individuals are better equipped to step into each other’s roles when necessary.

In this paper, we propose that humans facilitate efficient role switching by spontaneously attending to and representing the goals of their co-actors.

### 1.2. Literature Review

From a cognitive perspective, mutual commitment to a shared goal is the basis of multi-agent cooperation. Representing others’ goals ensures the fast convergence toward a collective goal for multiple co-actors, as simulated in the “imagined-we” model. It also serves as preparation for potential future role replacements. In social coordination, participants typically take on complementary roles and form expectations about how these roles ought to be fulfilled (Grosse et al., 2010). By understanding and anticipating the goals of others, individuals can more easily adapt to taking over another’s tasks if needed.

There are other cognitive benefits to representing others’ goals. First, it aids in predicting their actions, reducing cognitive load and minimizing the constraints of collaboration. More efficient coordination is possible when individuals can predict teammates’ actions, allowing them to adjust their own behavior accordingly (M. E. Bratman, 2022). Moreover, these predictions tend to be forward-looking rather than reactive. This proactive approach to collaboration conserves cognitive resources, avoiding the need for constant inferential reasoning based on observed actions, which can be complex and computationally demanding.

In addition, recent studies have demonstrated a joint memory effect, where participants show preferential encoding of partner-relevant items and better memory for them across different types of tasks (e.g., motor tasks (Wagner et al., 2017), non-motor tasks (Elekes & Sebanz, 2020), conflict tasks). In a social-epistemic framework, it is believed that humans are motivated to represent the mental states of others, which enhances the success of future interactions (Fiske & Taylor, 2020; Thornton et al., 2019). This account is closely related to Theory of Mind (Premack & Woodruff, 1978), positing that joint memory effects are driven by a basic motivation to represent others’ mental states and monitor their knowledge.

However, it is important to note that the effects of collaboration on memory are not uniformly facilitative. A substantial body of research has documented a phenomenon known as collaborative inhibition, whereby individuals recalling information in groups often perform worse than the pooled performance of the same individuals working alone (e.g., Basden et al., 2000; Weldon & Bellinger, 1997). This effect is typically attributed to retrieval disruption, as individuals’ idiosyncratic retrieval strategies are interfered with by the presence of others.

At first glance, collaborative inhibition appears to contradict findings from the joint memory effect, which suggests that collaboration enhances memory for partner-relevant information. However, these two lines of research may reflect distinct underlying mechanisms operating at different stages of memory processing. Collaborative inhibition is primarily observed in explicit retrieval contexts, whereas the joint memory effect emerges during encoding, when attention is guided by a partner’s task or goals.

From this perspective, collaboration does not exert a uniform effect on memory; rather, its impact depends on how social information is integrated into cognitive processing. The present study builds on this distinction by examining whether, in a cooperative context characterized by shared goals, attention and encoding are preferentially allocated to partner-relevant information, thereby producing a memory advantage.

### 1.3. Current Study

These findings provide a cognitive foundation for understanding how humans engage in efficient cooperation and rapidly substitute the tasks of others.

The present focus on representing a partner’s goals is closely related to the broader literature on action corepresentation in joint action research. For example, studies using the joint Simon task have shown that individuals spontaneously represent their partner’s actions at the level of stimulus–response mappings, even when those actions are not required for their own task (Sebanz et al., 2003, 2005). Such findings suggest that, in joint contexts, individuals integrate aspects of their partner’s task into their own cognitive system.

However, most prior work has focused on action-level representations, such as motor responses or stimulus–response associations. In contrast, the present study examines whether individuals represent their partner’s goals at a more abstract level. Compared to action representations, goal representations may play a more central role in structuring cooperative behavior, as they capture the functional organization of the joint task and support coordination across time, particularly in situations involving role differentiation and switching.

In addition, existing studies have not examined whether partner-goal representations formed during real-time joint action are retained over time. Most experiments were completed in a single session, with few studies conducting task-switching tests after the first session. Consequently, we have limited knowledge about participants’ long-term memory performance regarding others’ goals and whether this information is retained in long-term memory.

More relevant studies were conducted by He (He et al., 2011, 2014), combining working memory and attention guidance with surprise memory tests. In his study, two participants seated side by side were tasked with searching for pictures from different categories, with a third category used as a baseline. At the start of each trial, the screen indicated who was responsible for the trial. The participant assigned to operate had to memorize whether the picture matched the assigned category. A visual search (VS) task followed, where the target picture could either be near the relevant (preceding) picture or an irrelevant (non-preceding) one. He found that when the pre-search picture was related to a partner’s goal, it served as a cue that guided the subsequent search. Critically, during the surprise memory test, participants recognized more pictures related to the other’s category than to the baseline category. He’s studies suggest that others’ goals can automatically guide attention and influence memory encoding. However, He’s experiments involved participants performing tasks separately, which allowed time for processing task-irrelevant information, including the goals of others. Therefore, it primarily focused on two individuals completing their tasks independently, without examining joint action in real-time collaborative situations.

To address these gaps, this study examines whether people spontaneously encode and retain information about a partner’s goals, and whether this memory bias facilitates future role switching in cooperative tasks. Specifically, we investigate whether goal-related memory remains intact over time and how it supports role flexibility in ongoing collaboration. To address this, we devised a new joint search paradigm to test whether individuals spontaneously represent and preferentially encode others’ goals. In this paradigm, each participant in a dyad is assigned to search for items from a specific category while collaborating with another participant. To foster cooperation, task success is defined as both participants successfully detecting the target items. Following the search task, participants are tested with a surprise recall task, where they are required to identify whether they had seen specific items during the search.

A critical issue is how to distinguish shared intentionality from alternative, lower-level explanations such as semantic priming, stimulus salience, or passive task-set activation. These alternative accounts predict that partner-related stimuli may receive additional processing simply because they belong to a relevant category, are perceptually salient, or are associated with an activated task rule. However, they do not require that individuals represent the partner’s goal as part of a jointly structured action.

In contrast, shared intentionality involves representing a partner’s goal as functionally integrated with one’s own goal within a “we”-based framework. In the present paradigm, this is supported by the interdependence of task success: a trial is considered successful only when both participants respond correctly. This manipulation creates a cooperative structure in which the partner’s goal is not merely observed, but is relevant to achieving the joint outcome.

To test this, we operationalize spontaneous representation of a partner’s goal in two ways. First, at the level of attention, we examine whether the presence of a partner-relevant target—despite being irrelevant to the participant’s own task—affects search performance, as reflected in increased processing time. Second, at the level of memory, we assess whether partner-relevant items are preferentially encoded, as indicated by enhanced recognition accuracy in a surprise memory test. Together, these measures allow us to examine whether partner-related information is spontaneously integrated into cognitive processing in a way consistent with shared intentionality.

We hypothesized that representations of and memory enhancements for others’ goals would occur across different types of search categories (Expt. 1: animate items; Expt. 2: inanimate items). In a subsequent task, participants were required to switch search categories after a 3-day delay (Expt. 3). Our findings suggest that the memory performance for items related to others’ goals is maintained over time, and this effect extends to self-performance enhancement.

## 2. Expt. 1 Animate Materials

Experiment 1 aimed to investigate the spontaneous representation and preferential memorization of a partner’s goals. We built upon the classical visual search paradigm by adapting it into a collaborative context. In this joint search task, pairs of participants were required to search for targets together, with the task being deemed complete only when both individuals provided correct responses. The spontaneous representation of the partner’s goal was assessed by comparing reaction times between conditions where partners’ targets were present and those where they were absent. This comparison revealed whether participants spontaneously represent their partner’s goal, even when not explicitly required to do so, and whether their own search behavior is influenced by the presence of the partner’s targets. Additionally, a surprise recognition (SR) task followed the joint search, designed to assess memory enhancement for the partner’s goal. This task aimed to determine whether participants showed a bias toward memorizing their partner’s targets over other irrelevant items.

### 2.1. Methods

#### 2.1.1. Participants

Fifteen pairs of participants (*N* = 30, 10 men, *M*_age_ = 20.07, *SD*_age_ = 1.82) from Zhejiang University were recruited for credits or monetary payment (1 point/60 min for credits and 30 Yuan/60 min for cash). The sample size was determined based on the classical study of the Joint Memory Effect (*N* = 30, Elekes & Sebanz, 2020). All participants were right-handed and had normal or corrected vision. All participants used the same keyboard. Participants provided informed consent to participate in the study. Given that the relationship between participants could potentially influence cognitive processes (Holmberg et al., 2018; Luchies et al., 2013; Richards et al., 2003), we ensured that participants in each pair had never met before the experiment. An additional analysis was conducted to examine the performance of pairs of participants of different genders (see Appendix A, Table A1). The analysis revealed that gender did not have an impact on the results. Consequently, in subsequent experiments, all participant pairs were matched by gender.

#### 2.1.2. Stimuli & Apparatus

**Figure 1:**
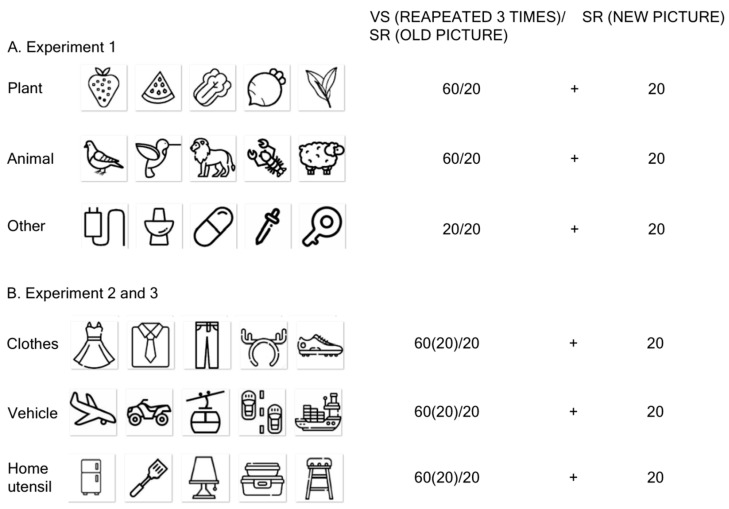
Illustration of the Stimuli Used in the Experiment. VS denotes a visual search task; SR denotes a surprise recognition test. 60(20)/20—the former number depends on whether the category is a target (60) or a distractor (20), and the latter number means how many pictures in the search task would be randomly selected for the recognition task.

Object images from different categories were used in the experiment. Targets consisted of two sets of 66 objects (Practice phase: 6, Formal phase: 60) representing either animals or plants. Distractors included object images from the following categories: clothing, furniture, necessities, and electrical appliances. In the practice phase, six pictures from each target category and 32 distractor images were presented (the distractor categories will be referred to as such for convenience in subsequent sections). In the formal phase, 60 pictures from each target category and 127 distractor images were used. All pictures were monochrome, devoid of anthropomorphic features (see Figure 1A), and sourced from two free design websites: https://www.iconfont.cn/ and https://www.flaticon.com/.

All stimuli were displayed on a 24-inch LCD monitor with a resolution of 1920 × 1080 pixels and a grey background (RGB: 128, 128, 128). The viewing distance was set to 60 cm. The experiment was conducted using Matlab 2014a with the Psychtoolbox package (Brainard, 1997; Pelli, 1997).

To ensure that the observed effects were not driven by low-level perceptual differences, we controlled stimulus properties across conditions. All images were monochrome icon-style drawings with comparable size, contrast, and level of visual detail. Old and new stimuli in the recognition test were drawn from the same semantic categories and were matched in terms of visual complexity and familiarity, as they were sampled from the same stimulus pool and design sources. Within each category, stimuli were selected to be representative and comparable in semantic typicality, avoiding highly distinctive or unusual items. In addition, the assignment of specific items to the “old” and “new” conditions was randomized across participants, further reducing the likelihood of systematic biases.

#### 2.1.3. Design & Procedure

**Figure 2:**
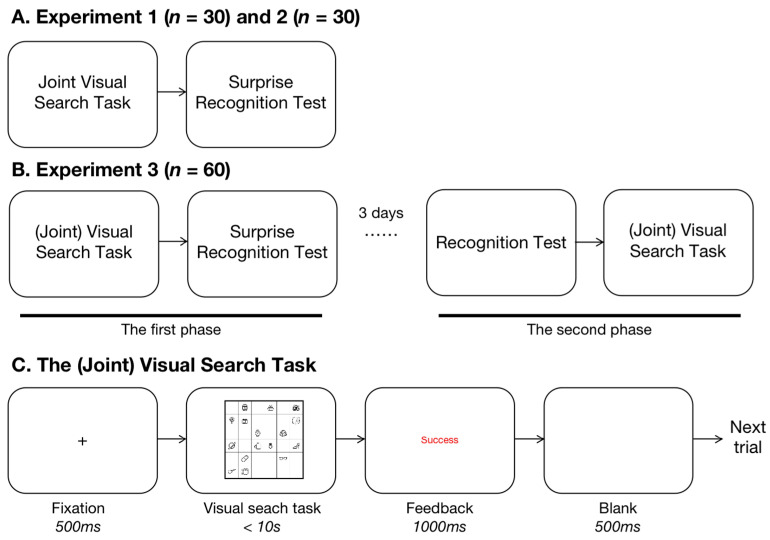
Illustration of the Procedure. (**A**,**B**) illustration of the process in the experiments. (**C**) *Joint search task*. A fixation was presented for 500 ms. A 6 × 6 white grid plane with 8, 16 or 32 items was displayed, during which participants needed to tell whether or not a target of the category assigned to them was on the plane. Participants’ responses followed feedback, which only suggested the success of the task when both of them had responded correctly.

The experiment was composed of two parts: the joint search task and the surprise recognition test (see Figure 2). All participants were informed that verbal/non-verbal communication was forbidden during the experiment.

*Joint search task*. We designed a joint search task in which performance depended on the responses of both participants, who were assigned to different and unrelated categories. Two categories (plants and animals) were randomly assigned to the participants, resulting in four distinct conditions based on target type: self-target (a target from the participant’s category, but no target from the partner’s category), partner-target (no target from the participant’s category, but a target from the partner’s category), both-target (a target from both the participant’s and the partner’s categories), and neither-target (no targets from either participant’s category). The set size in each trial was set to 8, 16, or 32 items, corresponding to the number of items in the visual search array. We hypothesized that participants would show longer reaction times (RTs) in the partner-target condition compared to the neither-target condition, indicating attention to the partner’s target.

Participants were seated side by side in front of the screen. The joint search task required both participants to provide correct answers, creating a cooperative setting. In each trial, a fixation cross was presented for 500 milliseconds, followed by a 6 × 6 matrix grid displaying object images in the center. Each object subtended a visual angle of approximately 2.65° × 2.65°. Participants were instructed to search for their own target and respond by pressing a designated key (‘A/D’ or ‘←/→’) to indicate whether a target was present or absent. The trial would continue until both participants responded, or the time limit of 10 s was reached. The task could only be considered successful if both participants responded correctly, and feedback was provided on the screen (either “the joint action was successful” or “the joint action was unsuccessful”). After feedback, a 500 ms blank screen was shown.

The practice phase consisted of 12 trials and ended when the accuracy rate exceeded 80%. In the formal experiment, each target picture appeared three times across target-present trials, leading to 180 trials with 60 different pictures. Including the target-absent trials, the formal experiment contained 360 trials, with each target-type condition having 90 trials (30 trials for each set size). Additionally, 20 distractor pictures were repeated three times for later recognition tasks. Breaks were provided at five intervals throughout the experiment. The total duration of the joint visual search task was approximately 30 min.

*Surprise recognition test*. A classical recognition memory test (Neath et al., 2003) was used in this study. The to-be-judged pictures belonged to one of three categories: the participant’s target category (Self), the partner’s target category (Partner), or the distractor category (Distractor) from the preceding joint search task. Each picture could either have appeared in that task (Old) or not (New). For the Old stimuli, 20 drawings from each category in the search task were selected, each of which appeared three times across trials. For the New stimuli, 20 completely new drawings from each category, which had never appeared in the previous search task, were used. This resulted in a total of 120 trials. Participants from each pair were assigned to separate rooms. In each trial, an object was presented at the center of the screen, which could either be an object presented during the joint visual search task or a new one. Participants were instructed to determine whether the object was one of the previously shown items (Old) or a new item (New), and respond by pressing the corresponding keys on the keyboard: “F” for Yes (Old) and “J” for No (New). The recognition task lasted approximately 5 min.

### 2.2. Results

In analyzing reaction times (RTs), all incorrect trials were excluded from the analysis and were not included in the report. For each participant, trials with RTs beyond three standard deviations from the mean in each condition were removed, resulting in the exclusion of 90 trials (0.883%) out of 10,195 data points.

**Errors.** The error rate for the joint search task was low and did not show significant differences between target-present conditions (self vs. both) or target-absent conditions (neither vs. other). These results were consistent across all experiments and will not be reported again (see Appendix A, Table A2).

**Figure 3:**
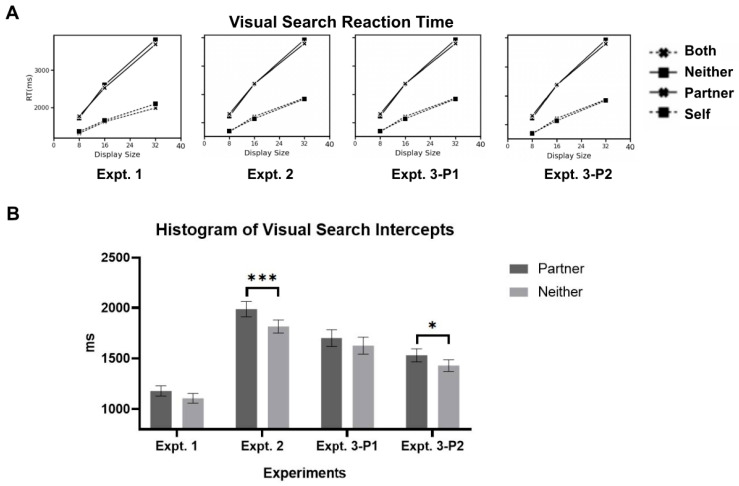
(**A**) Visual Search Reaction Time for Different Categories. (**B**) Histogram of Visual Search Intercepts. * *p* < 0.05, *** *p* < 0.001.

**Joint search RTs.** The search reaction times for the target-present conditions (self vs. both) and target-absent conditions (neither vs. partner) did not show significant differences. These results were consistent across experiments and will not be mentioned further in the text (see Appendix A, Table A3). Average intercepts and slopes of the RTs × set size were computed for each condition. The ratio of slopes between target-present and target-absent conditions was nearly 2:1 (see Appendix A, Table A3), suggesting that the search process is serial (Wolfe et al., 1992). The intercept reflects the delay in the onset of the search process or a delay in the post-search phase (e.g., choice selection or time to reject each distractor; Woodman et al., 2001). The difference in intercepts between the partner-target and neither-target conditions reflected the time spent on partner-relevant targets, either before or after the search. However, the intercepts for the partner-target and neither-target conditions did not differ significantly (*paired t-test*, *t*(29) = 1.717, *p* = 0.097, *d* = 0.273, *BF*_10_ = 0.717; partner-target: 1180 ms, neither-target: 1107 ms, see Figure 3).

**Figure 4:**
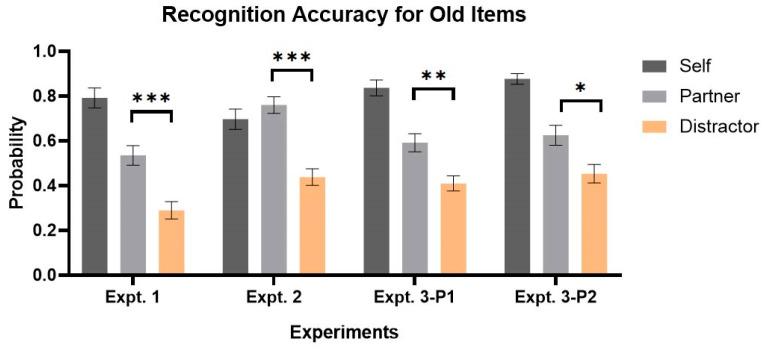
Recognition Accuracy for Old Items in All Experiments. * *p* < 0.05, ** *p* < 0.01, *** *p* < 0.001.

**Recognition accuracy.** Both the main effects of the target type and the item type were significant, together with a significant interaction effect (two-way ANOVA, target type: *F*(2, 58) = 68.00, *p* < 0.001, *η_p_*^2^ = 0.701, *BF*_10_ = 2.446 × 10^3^; item type: *F*(1, 29) = 24.84, *p* < 0.001, *η_p_*^2^ = 0.461, *BF*_10_ = 1.959 × 10^3^; interaction: *F*(2, 58) = 14.19, *p* < 0.001, *η_p_*^2^ = 0.328, *BF*_10_ = 13.11). Accuracy was higher for new items (*M_self_* = 0.748, *M_partner_* = 0.723, *M_distractor_* = 0.768) and did not show significant difference among conditions (*F*(2, 87) = 0.369, *p* = 0.692, *η_p_*^2^ = 0.008, *BF*_10_ = 0.038). Accuracy for old items across conditions showed significant difference (*M_self_* = 0.792, *M_partner_* = 0.535, *M_distractor_* = 0.290, *F*(2, 87) = 34.922, *p* < 0.001, *η_p_*^2^ = 0.445, *BF*_10_ = 2.301 × 10^2^, see Figure 4). Moreover, item recognition performance of partner’s target was better than that of distractors (*paired t-test*: *t*(29) = 5.65, *p* < 0.001, *d* = 1.189, *BF*_10_ = 4.714 × 10^3^). Other post hoc comparisons included: (Distractor vs. Self: *t*(29) = −10.30, *p* < 0.001, *d* = −2.369, *BF*_10_ = 2.817 × 10^8^; Partner vs. Self: *t*(29) = −6.78, *p* < 0.001, *d* = −1.178, *BF*_10_ = 8.124 × 10^4^).

### 2.3. Discussion

The critical finding of the study is that participants recognized the old items from their partner’s target category better than distractors. This result suggests a preferential encoding of partner-relevant items compared to distractors during the search task, even though the dyads had unrelated targets and could not influence each other’s search process. The phenomenon of participants recognizing new items better is understandable, as participants only encoded the old items for a brief period, potentially resulting in weak memory traces. Consequently, they may have developed a preference for categorizing those items as “new.” These critical findings, demonstrating preferential encoding of others’ targets, support the notion that people spontaneously represent others’ goals.

One consideration in the study pertains to the search categories—both target categories (animals and plants) are animate, which have higher perceptual salience and are therefore more likely to be detected quickly and accurately. Some might argue that the joint memory effect observed could be limited to these specific materials, with participants encoding only stimuli from their partner’s category when the targets are salient and easy to detect. To address this concern, we conducted Expt. 2 to test the effect using inanimate materials, to examine whether the results from Expt. 1 hold regardless of the visual properties of the materials.

## 3. Expt. 2 Inanimate Materials

The animal and plant images used in Experiment 1 possess salient characteristics of living things; such stimuli are more likely to capture attention (Jackson & Calvillo, 2013), making participants more sensitive to them. Therefore, in this experiment, stimulus including animals and plants with animate properties were excluded. The main purpose was to generalize the joint memory effect to other types of materials. We changed the target categories to three inanimate artifacts: vehicles, clothing, and home utensils (see Figure 1B), which represent basic natural categories (Rosch et al., 1976). Additionally, any two of the three artifact categories were randomly assigned to participants to eliminate the possibility that a specific category might play a key role in the effect. All other aspects of the experimental setup were the same as in Expt. 1.

### 3.1. Methods

#### Participants

Fifteen new pairs of naive participants of the same gender (*N* = 30, 12 men, *M_age_* = 20.30, *SD_age_* = 3.02) from Zhejiang University were recruited for course credit or monetary compensation (1 point/60 min for credits and 30 Yuan/60 min for cash). Participants in each pair had never met before the experiment. The sample size was determined to be the same as in Expt. 1.

### 3.2. Results

**Joint search RTs.** Significantly higher intercepts under the partner-target condition to the neither-target condition were observed (*paired t-test*, *t*(29) = 4.12, *p* < 0.001, *d* = 0.447, *BF*_10_ = 100.0; other-target: 1988 ms, neither-target: 1816 ms, see Figure 3).

**Recognition accuracy**. The results were consistent with those from Expt. 1. The main effect of target type and the interaction effect were significant (two-way ANOVA, target type: *F*(2, 58) = 21.66, *p* < 0.001, *η_p_*^2^ = 0.428, *BF*_10_ = 1.435 × 10^2^; item type: *F*(1, 29) = 2.07, *p* = 0.161, *η_p_*^2^ = 0.067, *BF*_10_ = 0.514; interaction: *F*(2, 58) = 9.42, *p* = 0.003, *η_p_*^2^ = 0.245, *BF*_10_ = 2.266). Accuracy was high for new items (*M_self_* = 0.717, *M_partner_* = 0.718, *M_distractor_* = 0.678) and did not show a significant difference among conditions (*F*(2, 87) = 0.347, *p* = 0.708, *η_p_*^2^ = 0.008, *BF*_10_ = 0.038). Accuracy for old items across conditions, however, showed significant differences (*M_self_* = 0.697, *M_partner_* = 0.760, *M_distractor_* = 0.438, *F*(2,87) = 18.17, *p < 0*.001, *η_p_*^2^ = 0.295, *BF*_10_ = 6.261). Furthermore, recognition performance for old items from the partner’s target was better than that for distractors (*paired t-test*: *t*(29) = 6.15, *p* < 0.001, *d* = 1.632, *BF*_10_ = 1.654 × 10^4^). Other post hoc comparisons included: (Distractor vs. Self: *t*(29) = −5.86, *p* < 0.001, *d* = −1.321, *BF*_10_ = 8.038 × 10^3^; Partner vs. Self: *t*(29) = 0.99, *p* = 0.328, *d* = 0.225, *BF*_10_ = 0.305).

### 3.3. Discussion

When stimulus animacy was removed, and the materials were replaced with entirely different artifact categories, the gap in recognition accuracy between new and old items was smaller than in experiment 1; Nevertheless, participants still showed better recognition for partner targets than for distractors. These results confirm our hypothesis that the representation and encoding of partners’ goals are stable across different types of materials. In addition, the intercepts in the partner-target condition were higher than those in the neither-target condition, suggesting that items belonging to the partner’s target category were spontaneously taken into account during the task.

We were further interested in whether the representation and memory of partners’ goals might confer additional advantages in subsequent interactions, particularly when the same individuals are required to cooperate again. Division of labor is a core component of human cooperation: the accomplishment of a joint intention involves coordinated actions in which collaborators assume complementary roles and responsibilities. However, individuals may not always be able to seamlessly perform their own roles without an understanding of the overall task structure. Representing others’ goals allows individuals to acquire knowledge about alternative subtasks and to monitor the global progress of the joint activity, rather than merely executing their own assigned subtasks.

This perspective aligns with Tomasello’s account of human cooperation, in which individuals adopt a “bird’s-eye view” of joint activities, enabling flexible role switching when necessary (Tomasello, 2014). From this standpoint, we examined whether dyadic behavior would differ when previously represented partner goals were reassigned as one’s own goals in a subsequent joint task. Information about a partner’s prior targets may be particularly useful when the same dyads reunite for further cooperation. Moreover, we were interested in whether the memory advantage for partner-relevant items persists beyond a single experimental session.

Accordingly, we propose two hypotheses. First, when a follow-up role reversal occurs, performance on tasks involving a partner’s former goals will be enhanced in a joint context. Second, when individuals anticipate future cooperation, the joint memory effect will be maintained over a longer period, thereby facilitating the successful implementation of subsequent cooperative actions.

## 4. Expt. 3 Three-Day Interval

Participants were recruited to attend the study sessions with a three-day interval. On the first day, they performed the same task as in Expt. 2, which aimed to test the stability of the results observed in the previous experiments. On the third day, participants returned to the laboratory to complete the same recognition task, followed by a joint search task that required them to switch the target categories. We focused on comparing performance differences in the visual task and recognition patterns for different categories across these two sessions, specifically examining the differences between conditions when participants completed the task individually versus dyadically.

### 4.1. Methods

#### 4.1.1. Participants

Fifteen new pairs of naive participants of the same gender (*N* = 30, 12 men, *M_age_* = 20.30, *SD_age_* = 3.02) from Zhejiang University were recruited for course credit or monetary compensation (1 point/60 min for credits and 30 Yuan/60 min for cash). Participants in Experiments 2 and 3 did not overlap with those in Experiment 1. Participants in each pair had never met before the experiment. The sample size was determined to be the same as in Expt. 1.

#### 4.1.2. Design & Procedure

The experiment was conducted in two phases (see Figure 2).

Phase 1: Participants completed a joint visual search task followed by a surprise recognition test. The experimental procedure was identical to that in Expt. 2, with the only difference being that participants were informed they would return to the laboratory in three days to complete a follow-up experiment (though the details were not provided at that time).

Phase 2: The same pairs of participants were invited back to the laboratory three days later. Each participant was taken to separate rooms to complete the same surprise recognition test as in Exp. 2. They were instructed to judge whether the items had appeared in the joint search task from three days prior. Afterward, the participants were brought into the same room to perform the joint search task again. The only change was that their target categories were swapped: participants were now required to search for the target category that their partner had searched for three days earlier. All other settings and equipment were consistent with Expt. 2.

### 4.2. Results

**Joint search RTs.** Significantly higher intercepts were observed in the partner-target condition compared to the neither-target condition in Phase 2 (Phase1: *paired t-test*, *t*(29) = 1.44, *p* = 0.161, *d* = 0.164, *BF*_10_ = 0.493; partner-target: 1701 ms, neither-target: 1626 ms; Phase2: *paired t-test*, *t*(29) = 2.63, *p* = 0.013, *d* = 0.307, *BF*_10_ = 3.514; partner-target: 1531 ms, neither-target: 1429 ms). Additionally, participants’ search reaction times were significantly shorter in Phase 2 (*paired t-test*, *t*(29) = 2.61, *p* = 0.017, *d* = 0.607, *BF*_10_ = 3.266).

**Recognition accuracy.** In Phase 1, the results were consistent with those in Expt. 2. The main effect of target type and the interaction effect were significant (two-way ANOVA, target type: *F*(2, 58) = 52.71, *p* < 0.001, *η_p_*^2^ = 0.645, *BF*_10_ = 1.867 × 10^5^; item type: *F*(1, 29) = 4.43, *p* = 0.044, *η_p_*^2^ = 0.132, *BF*_10_ = 1.540; interaction: *F*(2, 58) = 23.46, *p* < 0.001, *η_p_*^2^ = 0.447, *BF*_10_ = 2.423 × 10^2^). Accuracy was high for new items (*M_self_* = 0.753, *M_partner_* = 0.687, *M_distractor_* = 0.703) and did not show significant difference among conditions (*F*(2, 87) = 0.958, *p* = 0.388, *η_p_*^2^ = 0.022, *BF*_10_ = 0.046). Accuracy for old items across conditions showed significant difference (*M_self_* = 0.837, *M_partner_* = 0.592, *M_distractor_* = 0.410, *F*(2, 87) = 33.93, *p* < 0.001, *η_p_*^2^ = 0.438, *BF*_10_ = 1.902 × 10^2^). Furthermore, recognition performance for old items from the partner’s target was better than that for distractors (*paired t-test*: *t*(29) = 3.76, *p* = 0.002, *d* = 0.774, *BF*_10_ = 41.97). Other post hoc comparisons included: (Distractor vs. Self: *t*(29) = −9.43, *p* < 0.001, *d* = −2.071, *BF*_10_ = 4.261 × 10^7^; Partner vs. Self: *t*(29) = −6.66, *p* < 0.001, *d* = −1.178, *BF*_10_ = 6.100 × 10^4^).

In Phase 2, the main effect of target type and interaction were significant (two-way ANOVA, target type: *F*(2, 58) = 29.60, *p* < 0.001, *η_p_*^2^ = 0.505, *BF*_10_ = 1.275 × 10^3^; item type: *F*(1, 29) = 1.84, *p* = 0.185, *η_p_*^2^ = 0.060, *BF*_10_ = 0.459; interaction: *F*(2, 58) = 43.66, *p* < 0.001, *η_p_*^2^ = 0.601, *BF*_10_ = 3.209 × 10^4^). Accuracy was high for new items (*M_self_* = 0.712, *M_partner_* = 0.708, *M_distractor_* = 0.783) and did not show significant difference among conditions (ANOVA, *F*(2, 87) = 0.883, *p* = 0.417, *η_p_*^2^ = 0.020, *BF*_10_ = 0.045). Accuracy for old items across conditions showed significant difference (*M_self_* = 0.877, *M_partner_* = 0.625, *M_distractor_* = 0.453, *F*(2, 87) = 32.01, *p* < 0.001, *η_p_*^2^ = 0.424, *BF*_10_ = 1.305 × 10^2^). Furthermore, recognition performance for old items from the partner’s target was better than that for distractors (*paired t-test*: *t*(29) = 2.19, *p* = 0.037, *d* = 0.384, *BF*_10_ = 1.528). Other post hoc comparisons included: (Distractor vs. Self: *t*(29) = −6.60, *p* < 0.001, *d* = −1.450, *BF*_10_ = 5.165 × 10^4^; Partner vs. Self: *t*(29) = −5.86, *p* < 0.001, *d* = −0.906, *BF*_10_ = 8.021 × 10^3^).

### 4.3. Discussion

In Phase 1, we replicated the results of Expt. 1 and Expt. 2: participants exhibited better memory performance and showed improved recognition for partner-relevant items compared to distractors. The shorter reaction times in Phase 2 indicate that, after the target categories were switched between participants following a three-day interval, their search efficiency increased.

A potential limitation of Experiment 3 concerns the temporal order of tasks on day 3. Participants completed the recognition test prior to the second joint search task, which may have reactivated previously encountered stimuli and introduced a short-term priming effect. As a result, the improved search efficiency observed in the second joint task cannot be unambiguously attributed to long-term memory retention alone.

Importantly, however, the recognition results themselves provide independent evidence for the persistence of partner-related information over time. Participants showed enhanced recognition for partner-relevant items after a three-day interval, before re-engaging in the joint search task. This suggests that partner-goal information was retained in memory beyond the initial encoding phase.

Therefore, the facilitation observed in the second joint search task is best interpreted as reflecting the combined influence of previously encoded information and its potential reactivation. Rather than isolating a pure long-term memory effect, Experiment 3 demonstrates that partner-related information, once encoded, remains available and can be flexibly utilized in subsequent cooperative contexts.

Another concern is whether the observed memory advantage for partner-related items can be explained by category-level semantic priming, given that target categories were explicitly assigned. While semantic activation may contribute to processing efficiency, it is unlikely to fully account for the present findings.

If the effects were driven solely by category-level priming, similar memory advantages would be expected for both self-related and partner-related categories, as both were explicitly task-relevant. However, across experiments, we consistently observed differential patterns between self, partner, and distractor conditions, indicating that the effect is not reducible to general category activation.

Moreover, the critical manipulation in the present paradigm is not merely category membership, but the assignment of that category to a co-actor within a cooperative structure. The consistent advantage for partner-relevant items suggests that participants encode information in relation to their partner’s role, rather than solely as category members. This interpretation aligns more closely with accounts emphasizing the representation of others’ task-relevant goals in joint action.

The most notable finding is that participants could still recognize partner-relevant items more effectively, even after a three-day gap and task switching. These results suggested that the enhanced memory for partner-relevant items persisted beyond the completion of the related joint task, and this effect was accompanied by improved performance in the joint task. This efficient task switching implied that participants not only actively represented their partner’s goals during joint action, but were also prepared to take over the partner’s tasks. According to Zhou et al. (2022), this phenomenon may occur because participants automatically adopt their partner’s perspective and feel a sense of obligation to help complete “undone” tasks, even in the absence of the co-actor for some period. Additionally, the end of an experiment conveyed a clearer signal of the conclusion of a task, compared to the sudden absence of a co-actor. The results of this experiment indicated that the effect of representing and remembering a co-actor’s tasks is considerably stronger. Participants likely internalized this through a shared “we-intention” during the joint action. This provided further evidence that, during joint action, humans might adopt a “bird’s-eye view” as a shared “we” agent, where each individual is replaceable, thus facilitating efficient cooperation.

## 5. Discussions

This study employed a joint visual search paradigm in a dyadic collaboration context. Participants’ attention and memory performance were assessed using a visual search task and a subsequent recognition task. The results revealed that, in joint action contexts, individuals attend to and represent their partner’s targets. As a consequence of this processing, individuals devoted additional time to their partner’s targets and retained a greater number of images associated with those targets. Notably, a collaboration task with a stranger lasting only half an hour could produce a memory advantage that persisted for at least three days. After three days, this memory advantage for partner-related targets facilitated faster performance during subsequent cooperative interactions.

### 5.1. Cognitive Mechanisms of Representing Co-Actors Goals

In the study by Eskenazi et al. (2013), the priority effect observed for partner-related memory was interpreted as a form of simulation of the partner’s action intentions. Individuals were assumed to enhance the encoding of task- or attention-related information associated with their partner in some manner. Specifically, individuals are able to represent actions observed or anticipated by their partner in the same way as their own actions; this form of representation essentially relies on the human motor system to simulate action (Sebanz et al., 2003). Evidence from event-related potential (ERP) studies suggests that such action simulation occurs when participants perceive stimuli that are targets for a co-actor, as reflected by larger P300 amplitudes under joint conditions (Sebanz et al., 2006).

However, Elekes and Sebanz (2020) argued that the aforementioned studies cannot establish a causal relationship between motor cortex activation and the elicitation of shared task representations; instead, motor cortex activation may merely be a correlational factor. In her work with colleagues (Elekes & Sebanz, 2020), an alternative explanation—the Social Epistemic Account—was proposed. According to this account, attending to and remembering a partner’s targets serves the purpose of mapping the partner’s knowledge state, reflecting a fundamental human motivation to track the cognitive states of task partners. If this hypothesis holds, joint memory effects should occur only when the information encoded is relevant to the participant’s task goals. More specifically, enhanced memory for a partner’s target words should be restricted to their semantic content rather than to perceptual features such as visual appearance (e.g., color). This prediction was supported by their findings.

In their study, a three-category Go/No-Go paradigm was employed, with words presented as stimuli, while task assignment rules were based on stimulus color. Participants were required to judge whether words presented in their own target color were positive or negative, rendering semantic processing incidental rather than task-mandated. The results showed that, in addition to a self-priority effect, a joint memory effect also emerged in the subsequent recall task. These findings demonstrate that when a task requires semantic processing of target stimuli, individuals exhibit enhanced memory for the lexical content of their partner’s targets as well.

In the present study, our findings further support the theoretical accounts outlined above. Participants indeed demonstrated superior memory for images belonging to their partner’s target category, indicating that learning about a partner’s knowledge cannot be adequately explained by simple social imitation alone. In real-world environments characterized by repeated interactions and multi-round exchanges, such a bird’s-eye–level allocation of attention and memory to a partner’s targets may facilitate future cooperation. Through the continuous tracking of a partner’s knowledge state, individuals can further use this newly acquired information to construct shared knowledge between interacting agents, thereby giving rise to shared attitudes and shared beliefs. As the scale of cooperation expands, partner-specific knowledge systems may contribute to the formation of shared knowledge representations in repeated interactions, although this possibility requires further empirical investigation.

Notably, this form of social–cognitive processing may be characteristic of human cooperative behavior, although direct comparisons with other species are beyond the scope of the present study. Compared with other primates, humans engage in a much broader range of cooperative activities that are not restricted to kin-based interactions, and members of different cultural groups routinely cooperate in diverse activities. These activities rely on distinct cognitive skills, implying a high degree of flexibility in human cognition and a cultural environment that expects individuals to engage in flexible role differentiation across cooperative contexts. In a seminal study by Tomasello et al. (2005), researchers examined the social–cognitive abilities of three chimpanzees raised by humans. Although these chimpanzees performed similarly to human infants on relatively individualistic social–cognitive tasks, substantial differences emerged in cooperative tasks. For example, in a simple cooperative task in which a human participant assumed one role by lifting a tray while the chimpanzee performed the complementary role of placing a toy on it, the chimpanzees failed to perform the task once the roles were reversed, or simply disengaged from the interaction altogether. In contrast, in comparable experiments, human infants not only willingly switched roles but also actively anticipated their partner’s role change; when interacting with adults, they even engaged in expectant eye contact, signaling their understanding that the adult should continue performing the newly assigned role in the cooperative task (Carpenter et al., 2005).

One important question concerns whether the present findings can be explained by lower-level mechanisms such as semantic priming, stimulus salience, or passive task-set activation. While these factors may contribute to the processing of partner-related stimuli, they are unlikely to fully account for the observed pattern of results.

First, semantic priming or category-based activation would predict enhanced processing of all category-relevant items, but would not necessarily lead to selective memory advantages tied specifically to a partner’s role within a cooperative structure. Second, stimulus salience accounts would predict effects driven by perceptual features, whereas our results generalize across different types of materials (animate and inanimate), suggesting a more abstract mechanism. Third, passive task-set activation would not predict the persistence of partner-related memory over a three-day interval, nor its facilitative role in subsequent role switching.

In contrast, the shared intentionality account provides a more comprehensive explanation by positing that individuals represent their partner’s goal as part of a jointly structured task. This “we-representation” naturally leads to both enhanced encoding of partner-relevant information and its retention over time, as such information is functionally relevant for sustaining cooperative interaction.

### 5.2. Trade-Off Between Cognitive Resources and Cooperation Efficiency Implicates Automatic Representing of Co-Actors Goals

In the present study, one predictable outcome was that, if partner-related targets received additional processing, such processing would necessarily affect participants’ visual search performance under conditions of limited cognitive resources. The results of experiments 1–3 showed that when a partner’s search targets were present, the intercept of participants’ visual search functions was significantly higher. This finding indicates that a portion of the participants’ cognitive resources was indeed allocated to processing their partner’s targets.

Similar results have been reported in previous studies on dyadic visual search, particularly in tasks involving time pressure. Under conditions of limited cognitive resources, the coordination processes inherent in cooperation can themselves exert a negative impact on individual task performance. Although interpersonal cooperation is generally intended to improve task outcomes, the coordination required during cooperation entails corresponding costs for the individual (Brennan et al., 2008).

Converging evidence for such partner-related costs can also be found in the domain of memory. Recent research on social interaction and memory performance suggests that social context can modulate memory processing at multiple stages, ranging from initial encoding (Eskenazi et al., 2013) to post-encoding information exposure, as demonstrated in social contagion paradigms (Koppel et al., 2014; Meade & Roediger, 2002) and extending to retrieval processes (Bietti, 2010). Some of these social modulations exert detrimental effects on memory, including reduced accuracy, the formation of false memories, or impaired retrieval.

It is important to note that, although this section has discussed the potential costs associated with representing a partner’s task, the central issue is not whether such representation is ultimately beneficial or detrimental—an evaluation that, from an evolutionary perspective, would clearly favor its adaptive value—nor is it a matter of weighing its relative costs and benefits. Rather, the critical question concerns why humans are willing to incur such costs in order to represent their partner’s goals. One possible explanation is that attention to and memory for a partner’s targets operate in an automatic manner.

According to Bargh’s (2016) definition of automaticity, “automatic” processing in social cognition refers to the passive activation of internal mental representations. The defining feature is not the absence of consciousness per se, but rather individuals’ lack of awareness of how their behavior, attention, and judgments are being influenced. From this perspective, the representation of a partner’s targets observed in the present study qualifies as automatic social–cognitive processing. First, the visual search task never required participants to memorize any of the task stimuli. Second, when participants were informed after the search task that a subsequent image recognition task would follow, the majority expressed surprise and reported having forgotten all of the images; some even indicated after completing the recognition task that they had responded at random. Nevertheless, the experimental results showed that participants’ memory for their partner’s targets was reliably superior to baseline performance, despite their lack of awareness of this advantage. Thus, the representation of a partner’s targets appears to have been passively activated.

An important theoretical implication of the present findings concerns the seemingly inconsistent effects of collaboration on memory reported in the literature. While collaborative inhibition has been widely documented in group recall tasks, our results consistently demonstrate a collaborative advantage in the form of enhanced memory for partner-relevant information.

We propose that this discrepancy can be resolved by distinguishing between different stages and functions of memory. Collaborative inhibition primarily arises at the retrieval stage, where the presence of others disrupts individuals’ retrieval strategies. In contrast, the joint memory effect observed in the present study reflects a facilitation at the encoding stage, where attention is guided by a partner’s goals within a shared intentional framework. Thus, collaboration can impair or enhance memory depending on whether social interaction interferes with internal retrieval processes or supports goal-directed encoding. In cooperative contexts where individuals share a common goal, attending to and encoding a partner’s task-relevant information may serve an adaptive function by facilitating coordination and future role flexibility. The present findings therefore help reconcile conflicting results in the literature by showing that collaborative facilitation and inhibition are not contradictory, but instead reflect different cognitive mechanisms operating under different task conditions.

### 5.3. Evolutionary Advantages of Representing Co-Actors’ Goals

As described in the Introduction, attending to a partner’s targets constitutes a fundamental prerequisite for coordination among cooperating individuals. Interestingly, untrained individuals rarely become consciously aware of this form of attention in everyday life; it is only when social interaction is unexpectedly disrupted that its underlying role becomes faintly visible.

Humans are not only highly accustomed to attending to their partners’ targets but are also remarkably skilled at exploiting this capacity to guide others’ attention. Evidence from early developmental research further suggests that children are able to communicate by pointing, either to request that adults perform an action (imperative pointing) or to share experiences or emotions (declarative pointing) (Carpenter et al., 1998). This behavior exemplifies a strategic use of a deeply rooted social capacity: because humans are predisposed to attend to others’ targets, infants cleverly exploit this tendency to “recruit” adults into acting on their behalf. Related evidence can also be found in behavioral economics. In repeated (i.e., non–one-shot) public goods games, participants often recognize that there is no direct causal link between their own actions and those of others; nevertheless, they infer a probabilistic connection and treat it as sufficient justification for contributing. Participants believe that their own contributions can motivate others to contribute as well (Masel, 2007). Similar reasoning applies to real-world decisions such as voting in elections (Quattrone & Tversky, 1984). Although a single vote has an extremely low probability of influencing the outcome, individuals reason along the lines of “If I do not act, who will?” Despite the absence of a causal relationship between one person’s vote and others’ votes, people believe that their behavior can influence their peers.

This illusion of control is grounded in the assumption that others are constantly attending to the behaviors and attitudes of those around them, making it seem reasonable to attempt to influence others through one’s own actions. It is important, however, to distinguish these cases from the present study. In behavioral economics, such behaviors typically arise from deliberate and reflective reasoning. In contrast, in the present study—and more broadly in social interaction—individuals represent their partners’ tasks and goals automatically, as discussed in the previous section. Compared with the processes emphasized in behavioral economics, the cognitive mechanisms investigated here operate at a more fundamental level.

### 5.4. Implications for Future Studies

The present study examined attention to and memory for a partner’s targets in joint action, a process that represents a critical component of the mechanisms underlying coordinated action. Nevertheless, within the broad and multifaceted domain of human social interaction, joint action constitutes a complex phenomenon that cannot be fully captured by any single cognitive process. A comprehensive account must also incorporate related lines of research, including Theory of Mind (Baron-Cohen, 1991), perspective taking (Batson et al., 1997), intention inference (Goldman & Sripada, 2005), joint commitment (Gilbert, 2017), and social learning (Bandura, 1977). The contribution of the present study should therefore be understood as addressing one foundational element within a much larger theoretical framework. Moreover, research on human cooperative communication is inevitably embedded in long and indirect evolutionary trajectories, which current empirical approaches are only beginning to address.

From a theoretical standpoint, future research on the representation of partner-related goals should increasingly emphasize how humans construct a first-person plural, “we”-based perspective on the world. Rather than treating individual and joint representations as mutually exclusive, it is likely that human cognition flexibly integrates self-related (“I”) and collective (“we”) representations through both top–down and bottom–up processes, depending on contextual demands. Understanding how these representational levels are coordinated may prove crucial for explaining the efficiency and adaptability of human cooperation.

In addition, joint action should be conceptualized as a phenomenon that is not strictly bound to physical co-presence. While the present study focused on joint action within a shared spatial environment, the cognitive mechanisms supporting attention to and memory for a partner’s targets may extend to situations in which co-actors are spatially separated but psychologically connected through shared knowledge, intentions, or task awareness. Clarifying the boundary conditions under which joint action emerges will be essential for developing a more general theory of human cooperation.

Finally, the present findings should be interpreted within the scope of the experimental design. While we observed that partner-related information is preferentially encoded and retained over a short delay, such interpretations remain speculative and would require converging evidence from developmental, comparative, and cross-cultural research. Accordingly, the current study is best understood as providing evidence for cognitive processes that may support cooperation at the interpersonal level. Future research is needed to examine how these processes scale to more complex social systems and whether they generalize across different contexts and populations.

## Data Availability

All data and code can be found at https://osf.io/a8wtd/ (accessed on 8 February 2026). We used Python (version 3.8.3) to analyze the data. All statistical analyses were conducted using the Pingouin package (pg, version 0.4.5). For *t*-tests, we used the function “pg.pairwise_tests,” which directly provides Bayes factors. For ANOVAs, we used “pg.anova” for one-way ANOVA and “pg.rm_anova” for repeated-measures ANOVAs.

## Abbreviations

The following abbreviations are used in this manuscript:

VS	Visual Search
SR	Surprise Recognition
RT	Reaction Time

## Appendix A

**Table A1:** Search Accuracy and Reaction Time for Different Genders in All Experiments (Mean ± SEM).

Experiment	Gender	Search Accuracy	Reaction Time (ms)
Expt. 1	Female	0.946 ± 0.007	2211 ± 76
	Male	0.940 ± 0.009	2259 ± 98
Expt. 2	Female	0.841 ± 0.017	3438 ± 150
	Male	0.863 ± 0.016	3601 ± 167
Expt. 3 P-1	Female	0.870 ± 0.015	3336 ± 169
	Male	0.858 ± 0.019	3137 ± 180
Expt. 3 P-2	Female	0.890 ± 0.012	2854 ± 118
	Male	0.862 ± 0.023	2723 ± 224

**Table A2:** Search Accuracy in All Experiments (Mean ± SEM).

Experiment	Set Size	Self	Partner	Neither	Both
Expt. 1	8	0.947 ± 0.013	0.971 ± 0.007	0.994 ± 0.003	0.903 ± 0.017
	16	0.918 ± 0.016	0.977 ± 0.007	0.992 ± 0.003	0.891 ± 0.011
	32	0.886 ± 0.017	0.974 ± 0.006	0.988 ± 0.005	0.859 ± 0.017
Expt. 2	8	0.830 ± 0.019	0.930 ± 0.012	0.970 ± 0.006	0.822 ± 0.021
	16	0.801 ± 0.015	0.917 ± 0.013	0.941 ± 0.011	0.761 ± 0.023
	32	0.724 ± 0.023	0.894 ± 0.014	0.908 ± 0.014	0.701 ± 0.025
Expt. 3 P-1	8	0.878 ± 0.018	0.945 ± 0.009	0.967 ± 0.009	0.850 ± 0.016
	16	0.848 ± 0.020	0.917 ± 0.019	0.940 ± 0.018	0.813 ± 0.023
	32	0.742 ± 0.023	0.890 ± 0.031	0.885 ± 0.032	0.755 ± 0.028
Expt. 3 P-2	8	0.858 ± 0.023	0.953 ± 0.020	0.970 ± 0.014	0.848 ± 0.023
	16	0.798 ± 0.024	0.955 ± 0.019	0.953 ± 0.020	0.792 ± 0.022
	32	0.785 ± 0.020	0.898 ± 0.034	0.923 ± 0.027	0.752 ± 0.019

**Table A3:** Visual Search Intercepts and Coefficients in All Experiments (Mean ± SEM).

Experiment	Type	Self	Partner	Neither	Both
Expt. 1	Intercept (ms)	1126 ± 37	1180 ± 50	1107 ± 48	1117 ± 37
	Coefficient	31 ± 1	81 ± 4	86 ± 3	28 ± 1
Expt. 2	Intercept(ms)	1641 ± 79	1988 ± 76	1816 ± 65	1497 ± 70
	Coefficient	55 ± 3	110 ± 5	122 ± 5	58 ± 3
Expt. 3 P-1	Intercept (ms)	1393 ± 68	1701 ± 83	1626 ± 85	1567 ± 78
	Coefficient	56 ± 5	113 ± 7	115 ± 7	42 ± 6
Expt. 3 P-2	Intercept (ms)	1270 ± 51	1531 ± 64	1429 ± 57	1412 ± 59
	Coefficient	45 ± 6	87 ± 8	91 ± 8	35 ± 4

## References

[B1-behavsci-16-00690] Atmaca S., Sebanz N., Prinz W., Knoblich G. (2008). Action co-representation: The joint SNARC effect. Social Neuroscience.

[B2-behavsci-16-00690] Bandura A. (1977). Social learning theory. Canadian Journal of Sociology/Cahiers Canadiens de Sociologie.

[B3-behavsci-16-00690] Bargh J. (2016). Awareness of the prime versus awareness of its influence: Implications for the real-world scope of unconscious higher mental processes. Current Opinion in Psychology.

[B4-behavsci-16-00690] Baron-Cohen S. (1991). Precursors to a theory of mind: Understanding attention in others. Natural theories of mind: Evolution, development and simulation of everyday mindreading.

[B5-behavsci-16-00690] Basden B. H., Basden D. R., Henry S. (2000). Costs and benefits of collaborative remembering. Applied Cognitive Psychology: The Official Journal of the Society for Applied Research in Memory and Cognition.

[B6-behavsci-16-00690] Batson C. D., Early S., Salvarani G. (1997). Perspective taking: Imagining how another feels versus imagining how you would feel. Personality and Social Psychology Bulletin.

[B7-behavsci-16-00690] Bietti L. (2010). Sharing memories, family conversation and interaction. Discourse & Society.

[B8-behavsci-16-00690] Brainard D. H. (1997). The psychophysics toolbox. Spatial Vision.

[B9-behavsci-16-00690] Bratman M. (2014). Rational and social agency: Reflections and replies. Rational social agency: The philosophy of Michael Bratman.

[B10-behavsci-16-00690] Bratman M. E. (2022). Planning agency. The routledge handbook of philosophy of agency.

[B11-behavsci-16-00690] Brennan S. E., Chen X., Dickinson C. A., Neider M. B., Zelinsky G. J. (2008). Coordinating cognition: The costs and benefits of shared gaze during collaborative search. Cognition.

[B12-behavsci-16-00690] Carpenter M., Akhtar N., Tomasello M. (1998). Fourteen- through 18-month-old infants differentially imitate intentional and accidental actions. Infant Behavior and Development.

[B13-behavsci-16-00690] Carpenter M., Tomasello M., Striano T. (2005). Role reversal imitation and language in typically developing infants and children with autism. Infancy.

[B14-behavsci-16-00690] Cheng S., Zhao M., Tang N., Zhao Y., Zhou J., Shen M., Gao T. (2023). Intention beyond desire: Spontaneous intentional commitment regulates conflicting desires. Cognition.

[B15-behavsci-16-00690] Elekes F., Bródy G., Halász E., Király I. (2016). Enhanced encoding of the co-actor’s target stimuli during a shared non-motor task. Quarterly Journal of Experimental Psychology.

[B16-behavsci-16-00690] Elekes F., Sebanz N. (2020). Effects of a partner’s task on memory for content and source. Cognition.

[B17-behavsci-16-00690] Eskenazi T., Doerrfeld A., Logan G. D., Knoblich G., Sebanz N. (2013). Your words are my words: Effects of acting together on encoding. Quarterly Journal of Experimental Psychology.

[B18-behavsci-16-00690] Fiske S. T., Taylor S. E. (2020). Social cognition: From brains to culture.

[B19-behavsci-16-00690] Gilbert M. (2017). Joint commitment. The routledge handbook of collective intentionality.

[B20-behavsci-16-00690] Goldman A., Sripada C. (2005). Simulationist models of face-based emotion recognition. Cognition.

[B21-behavsci-16-00690] Grosse G., Moll H., Tomasello M. (2010). 21-month-olds understand the cooperative logic of requests. Journal of Pragmatics.

[B22-behavsci-16-00690] He X., Lever A. G., Humphreys G. W. (2011). Interpersonal memory-based guidance of attention is reduced for ingroup members. Experimental Brain Research.

[B23-behavsci-16-00690] He X., Sebanz N., Sui J., Humphreys G. (2014). Individualism–collectivism and interpersonal memory guidance of attention. Journal of Experimental Social Psychology.

[B24-behavsci-16-00690] Herrmann E., Call J., Hernández-Lloreda M. V., Hare B., Tomasello M. (2007). Humans have evolved specialized skills of social cognition: The cultural intelligence hypothesis. Science.

[B25-behavsci-16-00690] Holmberg M. J., Geri G., Wiberg S., Guerguerian A. M., Donnino M. W., Nolan J. P., Deakin C. D., Andersen L. W., International Liaison Committee on Resuscitation’s (ILCOR) Advanced Life Support and Pediatric Task Forces (2018). Extracorporeal cardiopulmonary resuscitation for cardiac arrest: A systematic review. Resuscitation.

[B26-behavsci-16-00690] Jackson R. E., Calvillo D. P. (2013). Evolutionary relevance facilitates visual information processing. Evolutionary Psychology.

[B27-behavsci-16-00690] Koppel J., Wohl D., Meksin R., Hirst W. (2014). The effect of listening to others remember on subsequent memory: The roles of expertise and trust in socially shared retrieval-induced forgetting and social contagion. Social Cognition.

[B28-behavsci-16-00690] Luchies L. B., Wieselquist J., Rusbult C. E., Kumashiro M., Eastwick P. W., Coolsen M. K., Finkel E. J. (2013). Trust and biased memory of transgressions in romantic relationships. Journal of Personality and Social Psychology.

[B29-behavsci-16-00690] Marean C. W. (2015). An evolutionary anthropological perspective on modern human origins. Annual Review of Anthropology.

[B30-behavsci-16-00690] Masel J. (2007). A Bayesian model of quasi-magical thinking can explain observed cooperation in the public good game. Journal of Economic Behavior and Organization.

[B31-behavsci-16-00690] Meade M. L., Roediger H. L. (2002). Explorations in the social contagion of memory. Memory & Cognition.

[B32-behavsci-16-00690] Mundy P., Newell L. (2007). Attention, joint attention, and social cognition. Current Directions in Psychological Science.

[B33-behavsci-16-00690] Muzzio I. A., Kentros C., Kandel E. (2009). What is remembered? Role of attention on the encoding and retrieval of hippocampal representations. The Journal of Physiology.

[B34-behavsci-16-00690] Neath I., Bireta T. J., Surprenant A. M. (2003). The time-based word length effect and stimulus set specificity. Psychonomic Bulletin & Review.

[B35-behavsci-16-00690] Pelli D. G. (1997). The VideoToolbox software for visual psychophysics: Transforming numbers into movies. Spatial Vision.

[B36-behavsci-16-00690] Premack D., Woodruff G. (1978). Does the chimpanzee have a theory of mind?. Behavioral and Brain Sciences.

[B37-behavsci-16-00690] Quattrone G. A., Tversky A. (1984). Causal versus diagnostic contingencies: On self-deception and on the voter’s illusion. Journal of Personality and Social Psychology.

[B38-behavsci-16-00690] Rekers Y., Haun D. B., Tomasello M. (2011). Children, but not chimpanzees, prefer to collaborate. Current Biology.

[B39-behavsci-16-00690] Richards J. M., Butler E. A., Gross J. J. (2003). Emotion regulation in romantic relationships: The cognitive consequences of concealing feelings. Journal of Social and Personal Relationships.

[B40-behavsci-16-00690] Rosch E., Mervis C. B., Gray W. D., Johnson D. M., Boyes-Braem P. (1976). Basic objects in natural categories. Cognitive Psychology.

[B41-behavsci-16-00690] Searle J. R. (1990). Collective intentions and actions. Intentions in communication.

[B42-behavsci-16-00690] Searle J. R. (1995). The construction of social reality.

[B43-behavsci-16-00690] Sebanz N., Bekkering H., Knoblich G. (2006). Joint action: Bodies and minds moving together. Trends in Cognitive Sciences.

[B44-behavsci-16-00690] Sebanz N., Knoblich G., Prinz W. (2003). Representing others’ actions: Just like one’s own?. Cognition.

[B45-behavsci-16-00690] Sebanz N., Knoblich G., Prinz W. (2005). How two share a task: Corepresenting stimulus–response mappings. Journal of Experimental Psychology: Human Perception and Performance.

[B46-behavsci-16-00690] Slors M. (2024). Explaining the cultural evolution of large-scale collaboration: Conventionality as an alternative for collective intentionality. Review of Philosophy and Psychology.

[B47-behavsci-16-00690] Tang N., Gong S., Zhao M., Zhou J., Shen M., Gao T. (2025). Joint commitment in human cooperative hunting through an “imagined we”. Proceedings of the Royal Society B: Biological Sciences.

[B48-behavsci-16-00690] Tang N., Stacy S., Zhao M., Marquez G., Gao T. (2020). Bootstrapping an imagined we for cooperation. Proceedings of the Annual Meeting of the Cognitive Science Society.

[B49-behavsci-16-00690] Thornton M. A., Weaverdyck M. E., Tamir D. I. (2019). The social brain automatically predicts others’ future mental states. Journal of Neuroscience.

[B50-behavsci-16-00690] Tomasello M. (2009). Why we cooperate.

[B51-behavsci-16-00690] Tomasello M. (2014). A natural history of human thinking.

[B52-behavsci-16-00690] Tomasello M. (2023). Differences in the social motivations and emotions of humans and other great apes. Human Nature.

[B53-behavsci-16-00690] Tomasello M., Carpenter M., Call J., Behne T., Moll H. (2005). Understanding and sharing intentions: The origins of cultural cognition. Behavioral and Brain Sciences.

[B54-behavsci-16-00690] Tomasello M., Vaish A. (2013). Origins of human cooperation and morality. Annual Review of Psychology.

[B55-behavsci-16-00690] Vesper C., Schmitz L., Safra L., Sebanz N., Knoblich G. (2016). The role of shared visual information for joint action coordination. Cognition.

[B56-behavsci-16-00690] Wagner U., Giesen A., Knausenberger J., Echterhoff G. (2017). The joint action effect on memory as a social phenomenon: The role of cued attention and psychological distance. Frontiers in Psychology.

[B57-behavsci-16-00690] Weldon M. S., Bellinger K. D. (1997). Collective memory: Collaborative and individual processes in remembering. Journal of Experimental Psychology: Learning, Memory, and Cognition.

[B58-behavsci-16-00690] Wolfe J., Friedman-Hill S., Stewart M. I., O’Connell K. M. (1992). The role of categorization in visual search for orientation. Journal of Experimental Psychology: Human Perception and Performance.

[B59-behavsci-16-00690] Woodman G. F., Vogel E. K., Luck S. J. (2001). Visual search remains efficient when visual working memory is full. Psychological Science.

[B60-behavsci-16-00690] Zhou J., Peng Y., Li Y., Deng X., Chen H. (2022). Spontaneous perspective taking of an invisible person. Journal of Experimental Psychology: Human Perception and Performance.

